# Experimental and Numerical Investigation of Slenderness Ratio on a Hollow Glued Bamboo Scrimber Column Under Eccentric Compression

**DOI:** 10.3390/ma19122508

**Published:** 2026-06-10

**Authors:** Yang Yang, Fuchun Li, Gang Yao, Lin Guo, Xian Yu

**Affiliations:** Key Laboratory of New Technology for Construction of Cities in Mountain Area, School of Civil Engineering, Chongqing University, Chongqing 400045, China; 20121601009@cqu.edu.cn (Y.Y.); 202416131175t@stu.cqu.edu.cn (F.L.); 202416021083@stu.cqu.edu.cn (L.G.); yuxian@stu.cqu.edu.cn (X.Y.)

**Keywords:** bamboo structure, hollow glued bamboo scrimber (HGBS), slenderness ratio, eccentric compression, numerical simulation

## Abstract

Hollow glued bamboo scrimber (HGBS), as a novel sustainable engineered bamboo material, exhibits considerable potential for structural engineering applications. To clarify the influence of slenderness ratio on the eccentric compression behavior of HGBS columns, an experimental and numerical investigation was conducted. A total of six HGBS specimens were tested under axial and eccentric compression to obtain their failure modes, load–displacement responses, and strain distribution characteristics. A detailed finite element model was developed in ABAQUS, in which bamboo scrimber was modeled as an orthotropic elasto-plastic material, while cohesive elements were employed to simulate the adhesive interfaces. The results indicate that HGBS columns subjected to eccentric compression exhibit pronounced axial force–bending moment interaction behavior. The average ultimate load under eccentric compression was only 17% of that under axial compression, demonstrating that the eccentric bending moment and second-order effects play a dominant role in reducing the load-carrying capacity. The finite element predictions agreed well with the experimental results, with deviations within 10%, confirming the reliability of the numerical model. Parametric analyses revealed that, as the slenderness ratio increased (corresponding to an increase in column height from 300 mm to 3000 mm), the ultimate load decreased from 104.17 kN to 28.20 kN, while lateral deformation and global instability became increasingly significant. The study elucidates the key influence of slenderness ratio on the eccentric compression performance of HGBS columns and provides a useful analytical basis for the design and application of engineered bamboo columns.

## 1. Introduction

In the context of the current low-carbon economic transition, the development of green and renewable construction materials has become a major trend in the building industry for environmental protection purposes [1,2]. Bamboo has drawn increasing attention due to its short growth cycle, abundant availability, strong renewability, and notable ecological benefits [3,4]. As a country rich in bamboo resources, China provides a solid material foundation for the large-scale application of bamboo in construction [5]. Although natural bamboo exhibits high tensile, compressive, and flexural strength, its limitations—such as restricted dimensions, irregular geometry, limited stability, and susceptibility to decay—hinder its direct use in structural engineering [6]. With advancements in bamboo processing technologies, engineered bamboo materials (such as bamboo scrimber), manufactured through fiber-bundle formation, gluing, and hot pressing, have overcome the shortcomings of raw bamboo while retaining the excellent mechanical properties of bamboo fibers [7,8]. Bamboo scrimber not only possesses high strength, good toughness, and stable performance, but also features low processing energy consumption and reduced environmental impact, offering superior mechanical properties compared with traditional timber [9,10]. Owing to these advantages, engineered bamboo materials have been applied in various construction projects worldwide and are regarded as promising alternatives to certain non-renewable building materials, providing a new pathway for promoting the sustainability of building structures [11,12].

At present, extensive research has investigated the mechanical performance of bamboo scrimber components subjected to axial loading [13,14,15], while recent studies have increasingly focused on the behavior of bamboo scrimber columns under eccentric loading. Li et al. [16] examined the influence of eccentricity on the eccentric compressive behavior of parallel bamboo-strip columns and reported that the load-bearing capacity decreased significantly with increasing eccentricity ratio, whereas deflection and strain first increased and then stabilized. An eccentricity influence coefficient was also proposed. Su et al. [17] conducted eccentric compression tests on glued laminated bamboo hollow columns and found that nonlinear material deformation and second-order effects substantially affected the ultimate capacity. Two analytical models for eccentric compression were established, along with a corresponding formula for predicting ultimate load. Zhao et al. [18] proposed a thin-walled steel tube–bamboo plywood hollow composite column with restraining bars and, through axial and eccentric compression tests, identified the effects of slenderness ratio, eccentricity, and bar arrangement on ultimate capacity. Xing et al. [19,20] developed a novel H-shaped steel–bamboo scrimber PEBS column and performed eccentric compression tests, demonstrating that the ultimate capacity could decrease by up to 70% as eccentricity increased, while ductility exhibited an initial increase followed by a decline. A predictive model for estimating ultimate capacity was also established. In summary, eccentricity is a key factor influencing the mechanical performance of bamboo scrimber columns. It not only governs the reduction in load-bearing capacity and the evolution of deformation but also significantly alters the failure modes and ductility characteristics of such components.

Regarding the mechanical performance of bamboo scrimber columns with different slenderness ratios, Chen et al. [21] investigated the axial compression behavior of bamboo scrimber columns with various slenderness ratios. Their results indicated that columns with larger slenderness ratios exhibited greater lateral displacement due to rapid development of lateral bending deformation and lower stiffness at early loading stages. Owing to instability-induced failure, the axial strain of the columns could not reach the ultimate strain of bamboo, preventing the material strength from being fully mobilized. Yu et al. [22] conducted axial compression tests on hollow glued bamboo scrimber columns with different slenderness ratios and found that, as the slenderness ratio increased, the failure mode transitioned from strength-controlled failure to instability failure. They also identified a critical slenderness ratio that governed the compressive capacity. Li et al. [23] carried out longitudinal compression tests on three groups of bamboo scrimber specimens with different slenderness ratios and observed that, at low slenderness ratios, the specimens tended to fail by compressive-shear failure. As the slenderness ratio increased, the dominant failure mode shifted to splitting failure. For specimens with the same slenderness ratio, compressive strength, peak strain, and elastic modulus decreased progressively as the specimen size increased. Janeshka et al. [24] further examined four commonly used test methods and design standards for engineered bamboo columns, specifically focusing on the recommended slenderness limits, and proposed a rational formula for estimating the compressive strength of intermediate-slenderness columns.

The above studies have deepened the understanding of the mechanical behavior of engineered bamboo structures and facilitated their development and application in practical engineering. However, existing research on hollow glued bamboo scrimber (HGBS) columns remains predominantly focused on their axial compression behavior. In real engineering scenarios, manufacturing and installation deviations often lead columns to experience eccentric compression, a loading condition that substantially increases the risk of structural failure. Therefore, systematically investigating the mechanical performance of HGBS columns under eccentric compression is of great significance for comprehensively understanding their structural behavior and guiding engineering practice. Nevertheless, despite its importance, research in this area is still relatively limited. In addition, slenderness ratio is another key parameter influencing the mechanical performance of HGBS columns. Hence, exploring the effect of slenderness ratio on the eccentric compressive behavior of HGBS columns holds essential theoretical significance and practical engineering value.

In this study, a combined approach of experimental testing and numerical simulation was adopted to systematically investigate the mechanical performance of HGBS columns under eccentric compression, with a particular focus on the influence of slenderness ratio on their load-carrying capacity and failure modes. A total of six HGBS column specimens were designed and fabricated for the experimental program, all with a cross-section of 100 mm × 100 mm, a panel thickness of 20 mm, and a length of 1200 mm. Depending on the loading eccentricity, the specimens were subjected to either axial compression tests or eccentric compression tests. The experiments provided the failure patterns, load–axial displacement responses, and strain distributions at different positions along the cross-section. A refined numerical model of the HGBS column was developed using the finite element software ABAQUS2025. By comparing the numerical predictions with the experimental results under both axial and eccentric compression conditions, the accuracy of the model was validated, after which a parameter analysis of slenderness ratio was conducted. Six column heights (300 mm, 600 mm, 1200 mm, 1800 mm, 2400 mm, and 3000 mm) were considered, corresponding to slenderness ratios (λ) of 8.9, 17.8, 35.6, 53.5, 64.9, and 89.1, respectively, with the eccentricity fixed at 90 mm for all models. The findings of this study provide a reliable analytical basis for evaluating and designing the load-carrying performance of engineered bamboo columns.

## 2. Materials and Test of HGBS Column

### 2.1. Specimens and Materials

**Figure 1:**
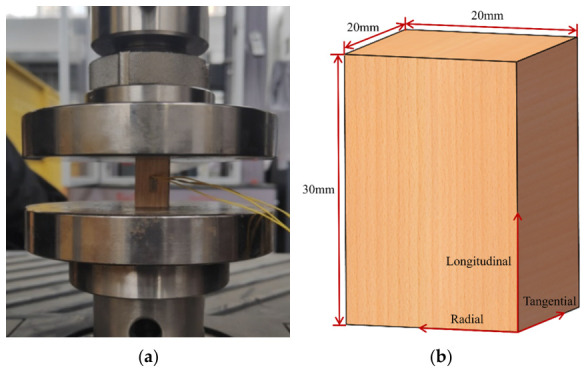
Material property compression test: (**a**) compression test; (**b**) dimensions of the compression test specimen.

The raw bamboo material used in this study was Neosinocalamus affinis harvested from Hongya County, Sichuan Province. Specimens with dimensions of 30 mm × 20 mm × 20 mm were prepared and tested in compression to determine their mechanical properties in accordance with the testing procedures specified in LY/T 3194 (2020) [25], as illustrated in Figure 1. Test results of material properties are shown in Table 1.

**Figure 2:**
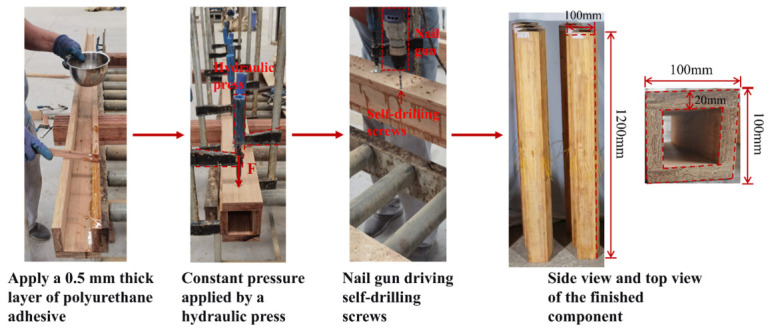
The production process and dimensions of the HGBS columns.

The HGBS columns were fabricated from four bamboo scrimber panels. During fabrication, the four panels were first polished and cut to the required dimensions. A uniform 0.5 mm layer of polyurethane adhesive was then applied to all contact surfaces, followed by 8 h of natural curing under hydraulic pressurization to ensure optimal interfacial bonding strength. Finally, self-tapping screws (M4.2  ×  32 mm) were driven in at 100 mm intervals using a nail gun to further enhance the mechanical interconnection between panels. The detailed fabrication procedure and the geometric characteristics of the cross-section are shown in Figure 2.

To investigate the mechanical performance of HGBS columns under eccentric loading, six HGBS column specimens were designed and fabricated. These specimens were divided into two test groups, each containing three identical samples and subjected to the same loading protocol to ensure statistical reliability. One group underwent axial compression testing, while the other group was tested under eccentric loading with an eccentricity of 90 mm. The load inclination angle for both groups was 0°. All specimens were produced with standardized dimensions: a length of 1200 mm, a cross-section of 100 mm × 100 mm, and a panel thickness of 20 mm. The cross-sectional configuration and dimensions of all test specimens correspond precisely to the finished side and top views shown in Figure 2. Table 2 presents detailed information on the test specimens, where “1200” indicates a specimen length of 1200 mm, “T20” indicates a panel thickness of 20 mm, and “0–0” denotes the eccentric angle and eccentricity.

### 2.2. Test Methods

**Figure 3:**
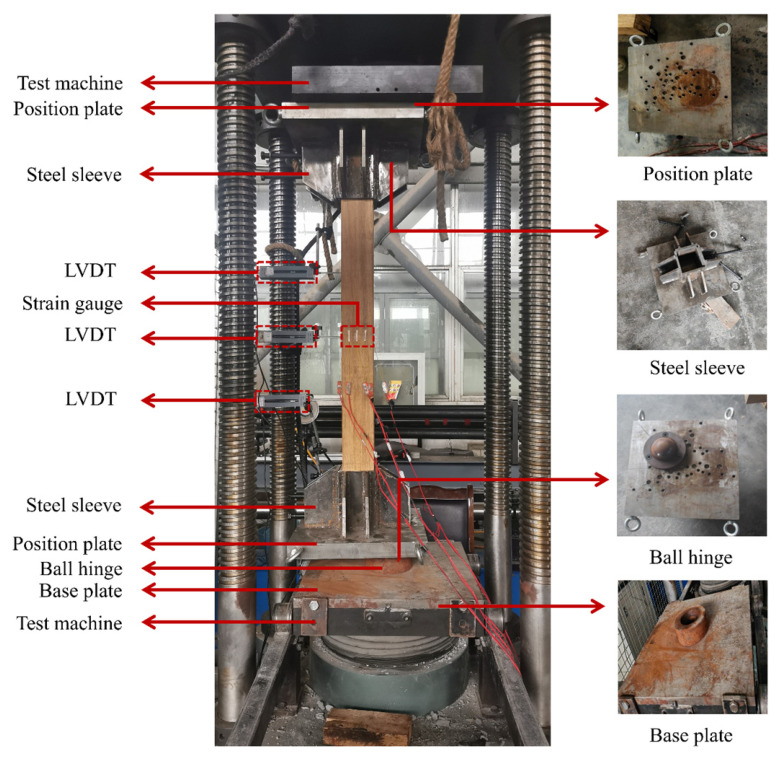
Eccentric Compression Experimental Setup.

The experimental program was carried out in the Civil Structures Laboratory on the Jiang’an Campus of Sichuan University. A microcomputer-controlled electro-hydraulic servo compression testing machine manufactured by Changchun New Testing Machine Co., Ltd. (Changchun, China) was used, and all tests were conducted in accordance with the Standard Test Methods for Timber Structures (GB/T 50329-2012) [26]. Prior to testing, crosshair reference lines were marked on both ends of each HGBS column to ensure geometric alignment during installation. To guarantee the accuracy of the loading position, a customized loading system was designed, consisting of a base plate, spherical hinge, positioning plate, and steel sleeves. All components were fabricated from Q345 structural steel. The configuration of the loading system is illustrated in Figure 3. The base plate serves as the primary connector between the loading device and the fixed spherical hinge. The spherical hinge, responsible for transmitting the applied load, was quenched during fabrication to improve stiffness and resistance to damage, preventing failure caused by stress concentration during loading. The positioning plate ensures accurate determination of the loading point. Steel sleeves with a height of 180 mm were installed at both ends of each HGBS column and equipped with adjustable bolts. After the specimen was positioned, the bolts on both sides were tightened to clamp and stabilize the column, preventing additional eccentric displacement during loading and avoiding any deviation of the loading point that could compromise data reliability. A spherical hinge integrated into the testing machine was mounted at the top of the loading system. To ensure precise alignment between the loading axis and the specimen axis, a laser level was used before loading to project a vertical reference line, enabling accurate calibration of the column’s verticality and minimizing potential errors caused by axial misalignment.

**Figure 4:**
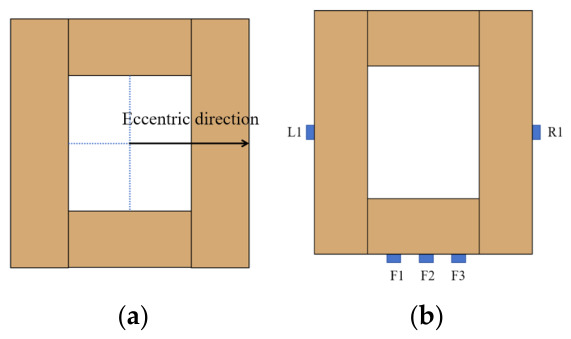
Direction of eccentric load and strain gauge positioning: (**a**) direction of eccentric load; (**b**) strain gauge positioning.

For the eccentric compression tests, the direction of the applied eccentric load on the HGBS columns is illustrated in Figure 4a. A YWD-200 linear variable differential transformer (LVDT) (Liyang Jincheng Testing Instrument Factory, Liyang, China) was installed along the height of each specimen to measure lateral deflection. Three 120-50AA strain gauges were mounted on the front face of the column, and one strain gauge was placed on each of the left and right faces to monitor the strain distribution along the column height during loading, as shown in Figure 4b. Throughout the testing process, strain and displacement data were continuously recorded using a TDS-530 data acquisition system.

Before the formal loading test, a preloading procedure was conducted at a rate of 5 kN/min to eliminate assembly gaps and any non-elastic deformations in the setup. The load was increased to 10 kN and then held for one minute, during which the strain responses recorded by the strain gauges on both sides were examined to verify the geometric alignment of the specimen. The formal loading process adopted a load–displacement hybrid control scheme. Load-controlled loading was applied first, with load increments set at 10% of the theoretical ultimate load, corresponding to 15 kN per increment, at a loading rate of 15 kN/min. Each increment was maintained for one minute. When the applied load reached 70% of the theoretical ultimate load, the loading mode was switched to displacement control, and the specimen was loaded at a rate of 2 mm/min until failure.

## 3. Experimental Results of HGBS Column

### 3.1. Failure Modes of HGBS Columns

**Figure 5:**
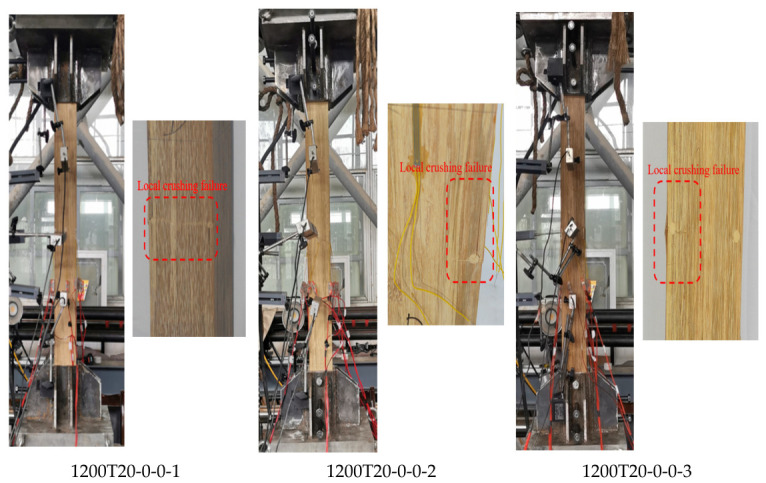
Failure modes of HGBS columns under axial compression.

**Figure 6:**
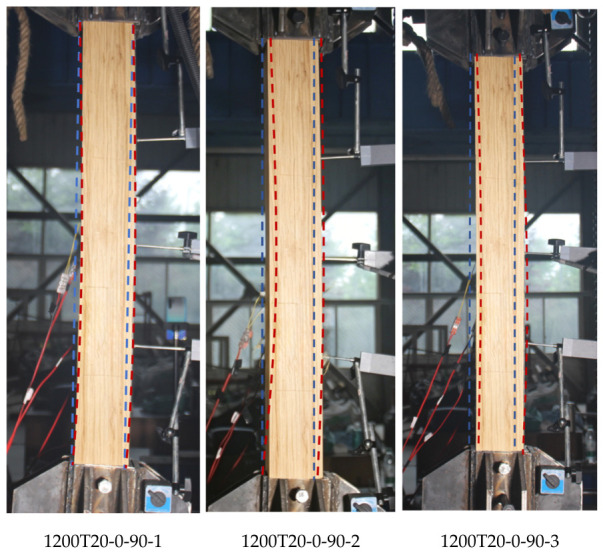
Failure modes of HGBS columns under eccentric compression. The blue dashed line represents the initial configuration of the HGBS column before loading, while the red dashed line represents the deformed configuration of the HGBS column at the ultimate state under eccentric compression.

The six HGBS specimens were divided equally into two groups for axial compression and eccentric compression tests, respectively. The failure modes observed under axial compression and eccentric compression are shown in Figure 5 and Figure 6.

As shown in Figure 5, the 1200T20 HGBS column exhibited no noticeable lateral displacement after axial compression failure. With further load increase, local stress concentrations induced by sectional weakening led to localized crushing of the specimen. Near the edge of the column, particularly in regions adjacent to the self-tapping screws, the bamboo fibers experienced slight buckling and developed a coupled damage pattern characterized by the combined effects of local compression and wrinkling. This damage process manifested as fiber pulverization, brittle fracture, and interlayer separation, indicating that the failure was governed by the intrinsic material failure rather than global instability. Overall, the axial compression failure of the HGBS column was primarily controlled by local material strength degradation, with the damage confined to a limited region, representing a typical local strength–dominated failure mode.

As shown in Figure 6, after failure under eccentric compression, the 1200T20 HGBS column exhibited pronounced lateral deflection in both its upper and middle regions. The column axis deviated significantly from its initial vertical alignment and developed substantial bending deformation along the longitudinal direction of the member, accompanied by compression–wrinkling coupled damage of the bamboo fibers. This damage pattern was characterized by localized fiber compression, progressive wrinkling, and interlayer failure, ultimately leading to global bending instability. The overall failure mode was governed by stability failure dominated by global buckling, indicating that the material strength of the HGBS column was not fully utilized. This behavior reflects the continuous accumulation of lateral displacement amplified by second-order effects under eccentric compression until instability occurred. The observed failure mechanism aligns with the predictions of Euler buckling theory, demonstrating that the bending moment–axial force interaction induced by eccentric loading plays a dominant role in the final instability of the member.

As shown in Figure 5 and Figure 6, the test observations indicate that under axial compression, the end restraints of the loading setup effectively suppressed lateral deformation, enabling the HGBS column to fully develop its global load-carrying capacity during loading. As a result, the overall material utilization was significantly improved. The final failure was characterized by a local crushing mode governed by localized strength degradation. In contrast, under eccentric compression, the HGBS column exhibited a distinctly different failure mechanism, dominated by global instability. With the continuous increase in eccentric bending moment, the lateral deformation of the member accumulated progressively until instability occurred. Moreover, due to the lower compressive strength of bamboo fibers compared with their tensile strength, the failure process was also accompanied by crushing damage on the compressive side.

### 3.2. Load–Displacement Curve and Ultimate Load of HGBS Columns

**Figure 7:**
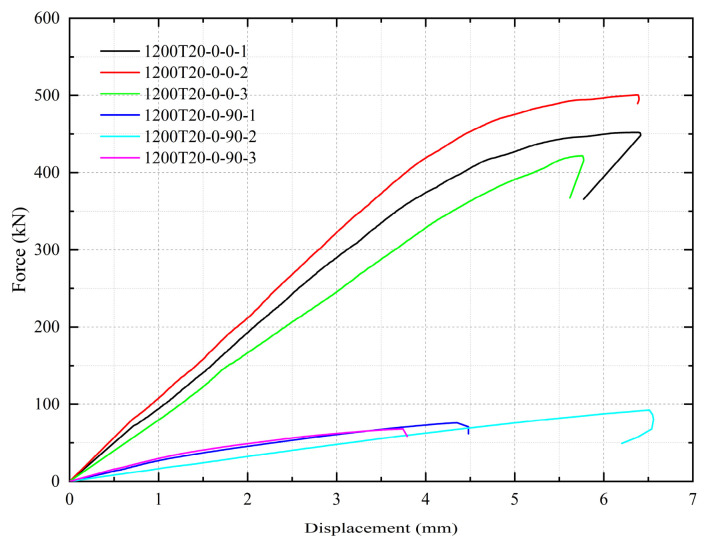
Load–axial displacement curves of HGBS column specimens under axial compression and eccentric compression.

The comparison of the load–axial displacement curves for the HGBS column specimens under axial compression and eccentric compression is shown in Figure 7. The peak load of the HGBS columns subjected to eccentric compression falls within the range of 70–100 kN, which is only about 20% of the ultimate load-carrying capacity of the axially loaded group. Moreover, the stiffness of the eccentrically loaded HGBS columns is significantly lower than that under axial compression. For the HGBS columns subjected to axial compression, the load–axial displacement curves exhibit three distinct stages: an elastic stage, an elasto-plastic stage, and a descending stage. In the elastic stage, the curve is linear, the structural integrity remains intact, and the member stiffness is stable. As the load increases, the displacement–load curve shows pronounced nonlinearity, where the load increases faster than the displacement, indicating the transition from the elastic stage to the elasto-plastic stage. During this stage, local damage begins to develop in the HGBS columns, such as outward bulging of compressed bamboo fibers. At the ultimate state, local crushing occurs near the column base, and a coupled failure mechanism involving fiber fracture, delamination, and crushing is observed. The curve then enters the descending stage, during which the load decreases; the lateral deformation of the HGBS column remains small, and axial deformation continues to dominate. Furthermore, the rapid post-peak strength degradation suggests that the failure was governed by material failure of the HGBS rather than global instability. In contrast, for the HGBS columns subjected to eccentric compression, the elasto-plastic stage is much shorter, accounting for only 10%~20% of the entire loading process. This is likely because the second-order effects amplified by the eccentric load intensify the lateral bending deformation, making the member more prone to global instability. As a result, the eccentrically loaded HGBS columns do not undergo sufficient internal force redistribution, causing the structure to reach instability and fail prematurely before its material strength can be fully mobilized. Moreover, the gradual post-peak decline in load-carrying capacity confirms that the failure was governed by global instability, preventing the bamboo scrimber material from fully developing its strength potential.

In addition, a certain degree of scatter can be observed in the load–displacement curves of replicate specimens under the same loading condition, particularly during the elastic stage. This is mainly attributable to the fact that HGBS is an engineered composite material manufactured from natural bamboo, in which variations in fiber distribution, density, and adhesive interface properties are unavoidable. Geometric imperfections introduced during specimen fabrication, as well as slight differences in the end-contact conditions of the HGBS columns during loading, may also influence the initial stiffness and deformation response of the specimens. For eccentrically loaded specimens, these effects are further amplified because the structural response is more sensitive to initial imperfections and additional eccentricities. Consequently, some variability is observed among the specimens during the elastic stage. To minimize the influence of initial imperfections on the experimental results, a laser level was used during the testing process to calibrate the vertical alignment of the specimens, thereby reducing installation errors and unintended additional eccentricity. Prior to formal loading, a preloading procedure was conducted to eliminate, as much as possible, any gaps between the loading system and the specimen, thereby improving the overall measurement accuracy of the tests. Despite these measures, minor initial imperfections were still unavoidable due to the inherent variability of natural bamboo-based materials and manufacturing tolerances. Nevertheless, good consistency is maintained in terms of failure modes, ultimate load-carrying capacities, and the overall evolution of the load–displacement curves. This indicates that the experimental results can reliably characterize the mechanical behavior of HGBS columns.

According to the ultimate bearing capacities of the HGBS columns listed in Table 3 under different loading conditions, the axial compression specimens reached ultimate loads of 451.98 kN, 500.54 kN, and 421.69 kN in the three tests, with an average value of 458.07 kN. This indicates that the specimens exhibited good stability in load-carrying performance under axial compression. In contrast, the eccentric compression specimens showed ultimate loads of 75.97 kN, 92.34 kN, and 68.01 kN, with an average of 78.77 kN, which is only 17% of the ultimate capacity of the axially compressed specimens. Overall, the test results demonstrate that eccentric compression significantly weakens the load-carrying capacity of HGBS columns. Compared with axial compression, the presence of eccentricity introduces coupling between bending moment and axial force, resulting in pronounced second-order effects as well as crushing and local instability on the compressed side of the column. Consequently, when the eccentricity increases from 0 mm to 90 mm, the ultimate capacity decreases sharply, and the failure mode shifts from local strength degradation to a moment–axial-force-dominated instability failure.

### 3.3. Strain of HGBS Columns

**Figure 8:**
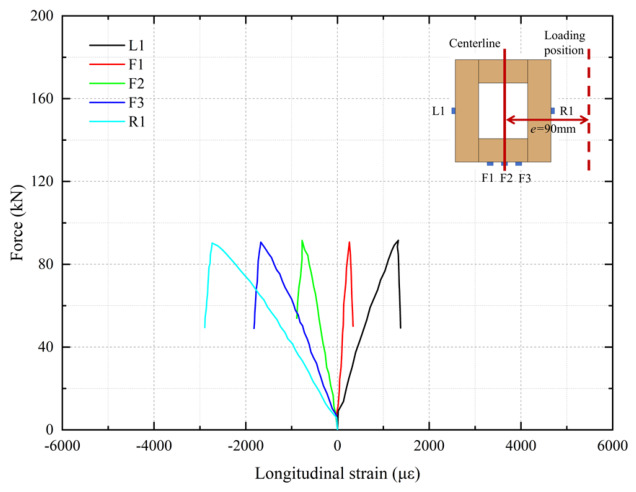
Load–strain curves of HGBS columns under eccentric compression.

As shown in Figure 8, the load–longitudinal strain curves of the HGBS column specimens under axial and eccentric compression are presented. The *Y*-axis denotes the applied load, while the *X*-axis represents the longitudinal strain recorded by the strain gauges. Two strain gauges were installed on the tension and compression sides of the HGBS column surface (R1 and L1), and three additional gauges (F1, F2, and F3) were uniformly arranged on the bamboo surface between them.

As shown in Figure 8, the strains recorded by the five strain gauges on the HGBS column specimens under eccentric compression include both positive and negative values. Depending on their stress states, the locations across the cross-sectional surface of the HGBS column experience either compression or tension. The load–axial strain curves of the HGBS columns under eccentric compression can be divided into three stages. The first stage is the elastic stage, during which the load–axial strain relationship is nearly linear as the load increases. The second stage is the elastic–plastic stage. In this stage, the slope of the curve decreases with increasing load, the growth rate of load reduces, and the relationship between load and strain becomes nonlinear. The third stage is the failure stage. Once the HGBS column reaches its ultimate state under eccentric axial compression, it immediately enters the descending branch. This phenomenon occurs because the specimen is simultaneously subjected to axial compression and bending moment. As the bending moment increases, lateral deformation becomes more pronounced. The presence of axial compression further amplifies the lateral deformation, which in turn magnifies the bending moment and accelerates the development of the lateral displacement. Consequently, the load-carrying capacity is rapidly lost, and the axial strain of the HGBS specimen cannot reach the material’s ultimate strain. This indicates that due to instability-induced failure, the material strength cannot be fully mobilized, ultimately leading to a failure mode dominated by global instability.

## 4. Numerical Model of the HGBS Column

### 4.1. Establishment of the Numerical Model

**Figure 9:**
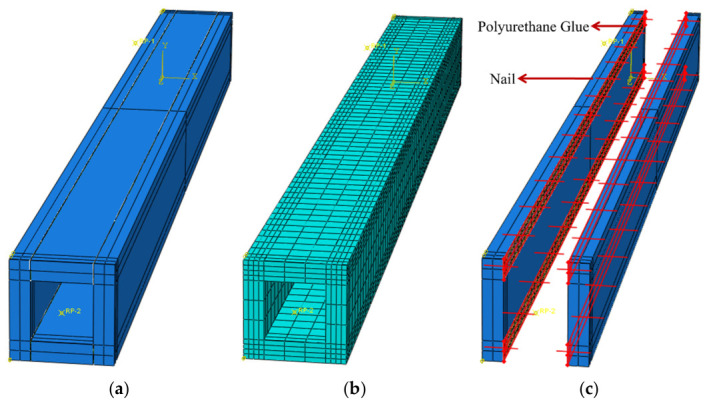
Finite element model of the HGBS column: (**a**) HGBS column model; (**b**) mesh configuration; (**c**) model of the adhesive layer and screws.

A finite element model (FEM) of the HGBS column was established using the ABAQUS finite element software, and its mechanical response was numerically analyzed with the ABAQUS/Standard implicit solver. To ensure comparability between the numerical results and the experimental conditions, the geometric dimensions, screw layout, and material parameters of the FEM were kept consistent with those of the test specimens. The meshing strategy and component models are shown in the figure, achieving a balance between computational accuracy and efficiency. The HGBS column and screws were modeled independently. The HGBS column was simulated using eight-node, three-dimensional reduced-integration solid elements (C3D8R), with element sizes controlled between 20 mm and 30 mm. The screws were modeled using two-node, three-dimensional truss elements (T3D2) and meshed with an element length of 1 mm. In total, the model consists of 9856 elements and 14,552 nodes. Figure 9 shows the complete finite element model and mesh of the HGBS column.

In the material definition module, the material properties of the HGBS column were defined as those of an orthotropic elasto-plastic material based on the material characterization tests described in Section 2.1 [27]. The mechanical behavior in both the elastic and plastic stages was characterized using the engineering constants listed in Table 1, thereby capturing the anisotropic characteristics of bamboo scrimber in different material directions. According to the results of the axial compression material tests on bamboo scrimber presented in Section 2.1, the yield stress and ultimate stress were specified as 77.8 MPa and 92.2 MPa, respectively, while the corresponding yield strain and ultimate strain were defined as 5.3 × 10^−4^ and 6.3 × 10^−4^. These parameters were used to describe the nonlinear response of the HGBS column after entering the plastic stage.

**Figure 10:**
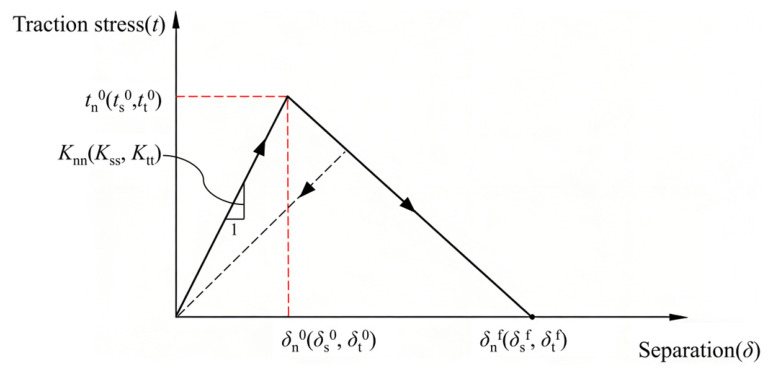
Typical traction–separation response.

The bonding layer, which serves as the interfacial connection between bamboo laminae, was modeled using cohesive elements to simulate the interaction between the bamboo scrimber and the adhesive. In ABAQUS, a bilinear traction–separation law was assigned to the cohesive material, and a cohesive section was created and applied to the interface elements consistent with the experimental configuration, enabling numerical simulation of the mechanical behavior of the bonding interface. The traction–separation constitutive relationship between the traction stress *t* and the relative displacement *δ* in the ABAQUS cohesive model is illustrated in Figure 10. The mechanical behavior of the bonding interface is characterized by defining the constitutive relationships between the relative displacement *δ* and one normal traction component (*t*_n_) together with two shear traction components (*t*_s_ and *t*_t_). The interfacial responses in the three directions are assumed to be independent and satisfy the linear elastic relationship: *t* = *Kδ*. Where *K* denotes the interface stiffness, including the normal stiffness (*K*_nn_) and the two tangential stiffnesses (*K*_ss_ and *K*_tt_). In the present study, the normal stiffness *K*_nn_ was taken as 3400 MPa/mm, while the tangential stiffnesses *K*_ss_ and *K*_tt_ were both assigned a value of 2000 MPa/mm.

When the resultant traction stress *t* formed by the stress components in the three directions reaches the peak traction stress *t*^0^, the relative displacement of the interface reaches the corresponding peak displacement *δ*^0^. At this point, damage initiation occurs at the bonded interface, and the interfacial stiffness begins to degrade progressively. As the relative displacement further increases to the failure displacement *δ*^f^, the traction stress decreases to zero, indicating a complete loss of bonding capacity. In the present study, the failure displacement *δ*^f^ was taken as 1 mm. The quadratic nominal stress criterion adopted for damage initiation is expressed as follows:

(1) $${\left(\frac{\langle t_{n}\rangle }{t_{n}^{0}}\right)}^{2}+{\left(\frac{t_{s}}{t_{s}^{0}}\right)}^{2}+{\left(\frac{t_{t}}{t_{t}^{0}}\right)}^{2}=1$$

where $t_{n}^{0}$, $t_{s}^{0}$, and $t_{t}^{0}$ denote the peak traction stresses in the normal and two tangential directions, respectively, all of which were taken as 5 MPa in this study. The symbol ⟨ ⟩ represents the Macaulay bracket, which ensures that damage initiation occurs only when the interface is subjected to tensile loading. Consequently, compressive stresses across the bonded surfaces do not contribute to damage evolution of the adhesive interface.

In the interaction and boundary condition settings of the finite element model, no relative slip was assumed between the screws and the HGBS column. The connection between the screws and the HGBS column was modeled using embedded region constraints, thereby accurately representing the force-transfer mechanism provided by the screws within the connection system [27]. For the HGBS columns subjected to uniaxial compression, reference points were assigned to the centers of the top and bottom surfaces, and these reference points were coupled with their corresponding end surfaces of the bamboo scrimber column. For the HGBS columns under uniaxial eccentric compression, the reference point was positioned at an eccentricity of 90 mm, consistent with the experimental configuration, and similarly coupled with the top and bottom surfaces. Finally, the boundary conditions of the finite element model were defined in accordance with the uniaxial compression tests of the HGBS columns described in Section 2.2. The translational displacements of the lower reference point were restrained in the X-, Y-, and Z-directions, while the translational displacements of the upper reference point were restrained in the Y- and Z-directions. A displacement-controlled load was then applied to the upper reference point in the X-direction.

### 4.2. Validation of the Numerical Model

To verify the accuracy and reliability of the established finite element model, a systematic comparison was conducted between the numerical simulations and the experimental results in terms of failure mode, load–displacement response, and ultimate bearing capacity. During the parametric analysis, only the HGBS columns height was varied to obtain different slenderness ratios, while the cross-sectional dimensions, screw spacing, adhesive layer modeling approach, material properties, loading scheme, load eccentricity, and boundary conditions were kept unchanged. This ensured that the observed differences in structural response could be attributed solely to the effect of the slenderness ratio, thereby guaranteeing the validity of the comparison.

#### 4.2.1. Comparison of Failure Modes

The finite element analysis adopted the S11 (longitudinal stress) contour as the primary stress evaluation index. Since HGBS is an orthotropic bamboo-based composite material whose principal load-carrying direction coincides with the longitudinal orientation of the bamboo fibers, the S11 stress component can directly reflect the stress distribution and stress concentration along the fiber direction. Compared with equivalent stress measures such as von Mises stress, which are primarily applicable to isotropic materials, S11 provides a more accurate representation of the stress state and failure mechanism of HGBS columns subjected to eccentric compression.

**Figure 11:**
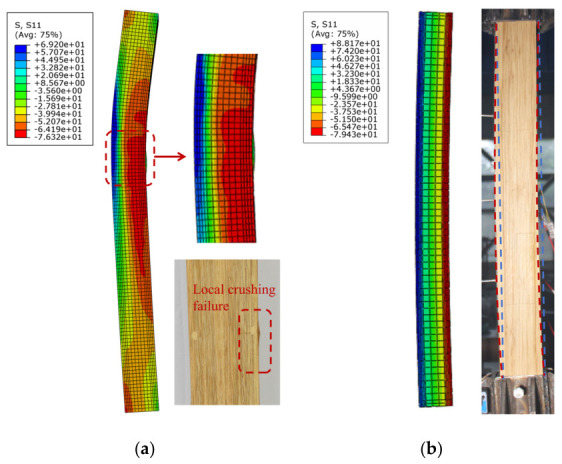
Comparison of final failure modes between experimental results and finite element analysis: (**a**) axial compression; (**b**) eccentric compression.

As shown in Figure 11a, the failure of the axially loaded specimens was mainly characterized by local crushing. The damage region was concentrated in the middle part of the column near the self-tapping screws, where the bamboo fibers exhibited compressive buckling, wrinkling, and interlayer separation. No significant global lateral deformation was observed. The finite element results show good agreement with the experimental observations. After reaching the ultimate load, the numerical model also exhibited local crushing, with the most severe damage occurring at the mid-height surface of the column. In addition, debonding damage was observed at the adhesive interfaces. No global buckling was predicted in the simulation, indicating that the model can accurately capture the local failure mechanism governed by material strength degradation under axial compression.

As shown in Figure 11b, the eccentrically loaded specimens exhibited significant global bending instability. The column axis clearly deviated from its initial vertical alignment, and the lateral deflection was mainly concentrated in the mid-height and upper-middle regions. Meanwhile, the crushing and wrinkling of bamboo fibers were observed on the compression side. The finite element simulation successfully reproduced this failure mode. During loading, lateral displacement gradually accumulated in the numerical model, ultimately leading to pronounced bending deformation in the mid-span region. The predicted global instability pattern was in good agreement with the experimental observations. The above comparison demonstrates that the proposed model can reasonably capture the dominant failure modes of HGBS columns under different loading conditions.

#### 4.2.2. Comparison of Load–Displacement Curves

**Figure 12:**
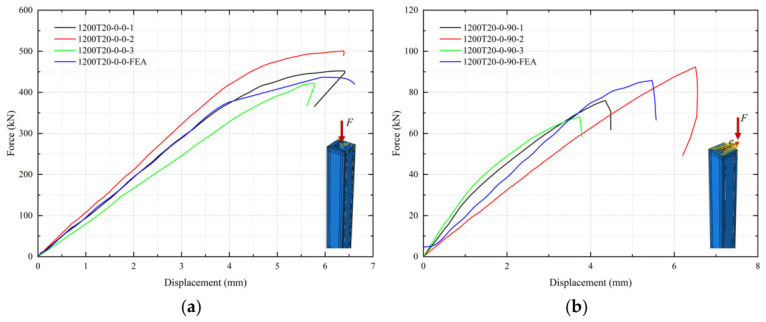
Comparison of load–displacement curves between numerical simulation and experimental results for HGBS columns: (**a**) axial compression; (**b**) eccentric compression.

Figure 12 shows the comparison of load–axial displacement curves between experimental results and finite element simulations of HGBS columns under axial compression and eccentric compression.

For both axial compression and eccentric compression cases, the stiffness in the elastic stage and the response in the elasto-plastic stage show good agreement between the numerical simulations and the experimental results. The ultimate load-carrying capacities predicted by the finite element model also match the experimental values well. The minor discrepancies between the finite element and experimental curves may be attributed to the fact that, in the actual specimens, the elastic modulus, yield strength, ultimate strength, and constitutive behavior of the material are not perfectly uniform along the cross-section and member length. Local weak regions may experience earlier damage or failure, thereby reducing the overall stiffness of the HGBS columns. In contrast, the finite element model typically adopts constitutive parameters based on averaged material test results and cannot fully capture such local weakening effects, leading to certain deviations between numerical and experimental results. Overall, the relative errors of the finite element analysis remain within an acceptable engineering range, indicating that the numerical model is reasonable and accurate and can reliably represent the load–displacement behavior of HGBS columns.

#### 4.2.3. Comparison of Ultimate Load-Carrying Capacity

The ultimate load-carrying capacities of the HGBS columns obtained from the experiments and finite element simulations are presented in Table 4.

For the axially loaded specimens, the ultimate load-carrying capacities obtained from the three tests were 451.98 kN, 500.54 kN, and 421.69 kN, respectively, with an average value of 458.07 kN. The finite element prediction was 436.50 kN, resulting in a relative error of 4.7% compared with the experimental average. For the eccentrically loaded specimens, the ultimate loads from the three tests were 75.97 kN, 92.34 kN, and 68.01 kN, respectively, with an average value of 78.77 kN. The corresponding finite element result was 85.81 kN, with a relative error of 8.9%. It should be noted that no additional initial geometric imperfections or eigenmode perturbations were introduced into the finite element model. Under eccentric compression, the numerical predictions are slightly higher than the average experimental values, which may be attributed to the presence of initial geometric imperfections and material heterogeneity in the actual specimens, leading to a reduction in their load-carrying capacity. Nevertheless, the discrepancy between the numerical and experimental ultimate loads remains within 10%, indicating that the proposed model can accurately capture the ultimate load-bearing behavior of HGBS columns subjected to eccentric compression.

Based on the comparative results in terms of failure modes, load–displacement response evolution, and ultimate load-carrying capacity, the developed finite element model of HGBS columns can accurately capture the overall mechanical behavior of the members under both axial and eccentric compression. The model demonstrates good reliability in predicting failure patterns, stiffness evolution, and load-bearing capacity, and satisfies the accuracy requirements for further parametric analyses. Therefore, it can be effectively used for subsequent systematic investigations into the influence of slenderness ratio.

### 4.3. Parametric Analysis of Slenderness Ratio

To further systematically investigate the influence of slenderness ratio on the mechanical performance of HGBS columns under eccentric compression, a parametric study was conducted based on the validated finite element model established above. The cross-sectional dimensions (100 mm × 100 mm, panel thickness 20 mm), eccentricity (e = 90 mm), and material parameters were kept constant.

The slenderness ratio (λ) of the HGBS column specimens can be calculated as follows:

(2) $$\lambda =\frac{l_{0}}{i}$$

(3) $$i=\sqrt{\frac{I}{A}}$$

where *l*_0_ is the effective length of the specimen; *i* is the radius of gyration of the cross-section; *I* is the second moment of area (moment of inertia) of the cross-section; and *A* is the cross-sectional area of the specimen.

**Figure 13:**
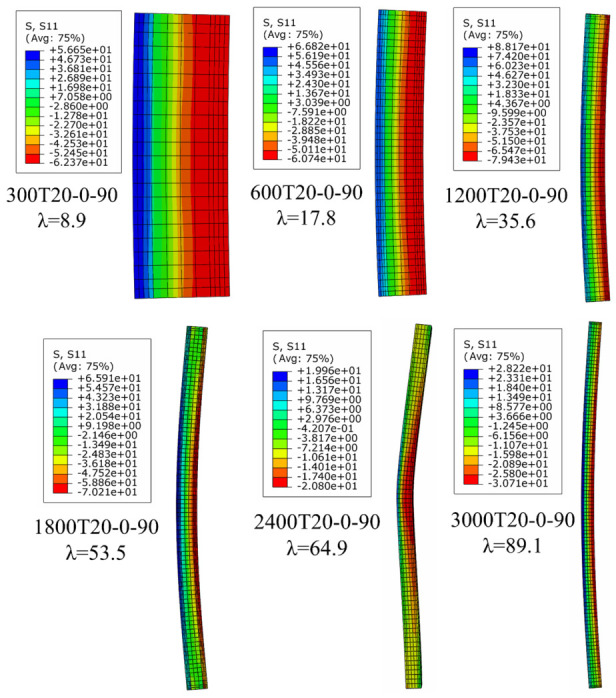
Failure modes of HGBS columns with different slenderness ratios under eccentric compression from numerical simulations.

Six different column heights were considered, namely 300 mm, 600 mm, 1200 mm, 1800 mm, 2400 mm, and 3000 mm, corresponding to slenderness ratios (λ) of 8.9, 17.8, 35.6, 53.5, 64.9, and 89.1, respectively. All models adopted the same pinned–pinned boundary conditions as in the experiments, and displacement-controlled loading was applied at the upper reference point through a coupling constraint. The failure modes of HGBS columns with different slenderness ratios under eccentric compression obtained from the numerical simulations are presented in Figure 13.

As shown in Figure 13, all specimens exhibited pronounced bending deformation during the loading process, and no failure due to local crushing was observed in short columns. In the initial loading stage, axial compression dominated the deformation behavior, with relatively small lateral deflection. As the load increased, noticeable lateral bending gradually developed in the mid-to-upper region of the columns, and this deformation became increasingly severe with further load increase. When the load approached the ultimate capacity, the lateral deflection increased rapidly, indicating a typical second-order effect associated with compression and bending interaction. Ultimately, all HGBS columns failed in a global instability mode, which can be attributed to stability loss induced by column flexibility, preventing full utilization of the material strength of bamboo scrimber. Under eccentric compression, all specimens exhibited evident lateral displacement, and the lateral bending deformation progressively intensified with increasing slenderness ratio. It should be noted that, for short columns with relatively low slenderness ratios (λ < 20), material failures such as local crushing would typically be expected to govern the structural response. However, due to the large eccentricity of 90 mm, a significant bending moment is introduced from the early stages of loading. For the HGBS columns considered in this study, the bending moment plays a more dominant role than the axial force in governing the structural behavior. Consequently, lateral deformation and second-order effects continue to develop throughout the loading process, ultimately leading to global instability failure in all specimens.

As shown in Table 5, when the column height is 300 mm (λ = 8.9), the ultimate load-carrying capacity is 104.17 kN. As the height increases to 600 mm (λ = 17.8), the capacity decreases to 95.08 kN, corresponding to a reduction of 8.7%. For a column height of 1200 mm (λ = 35.6), the capacity further decreases to 85.81 kN, which is 11.9% lower than that of the 300 mm column. When the height reaches 1800 mm (λ = 53.5), the capacity drops to 71.45 kN, representing a 31.4% reduction relative to the 300 mm case. For 2400 mm (λ = 64.9), the capacity sharply decreases to 40.58 kN, with a reduction of 61.0%. Finally, at 3000 mm (λ = 89.1), the capacity further declines to 28.20 kN, resulting in an overall reduction of 72.9%. These results indicate that the slenderness ratio has a pronounced nonlinear weakening effect on the eccentric compressive capacity of HGBS columns. In particular, when λ exceeds 60, the rate of capacity degradation increases significantly.

**Figure 14:**
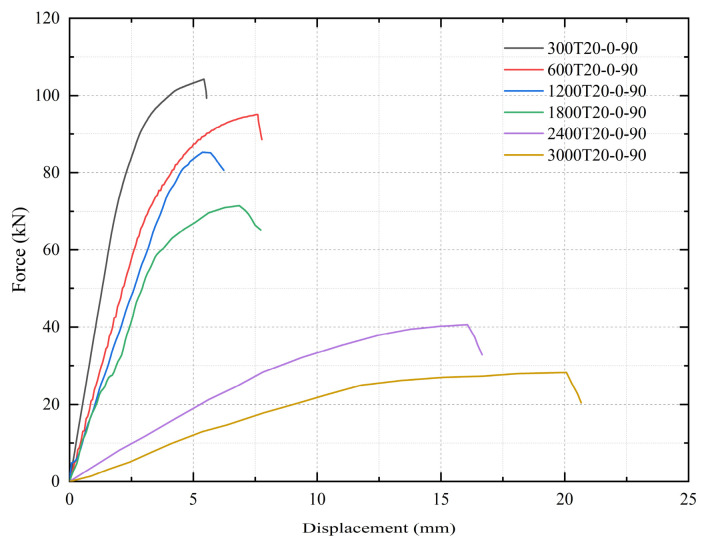
Load–displacement curves of HGBS columns with different slenderness ratios under eccentric compression.

From the evolution of the load–displacement curves in Figure 14, it can be observed that when the slenderness ratio is relatively small (λ < 20), the curves exhibit a steep slope in the elastic stage and a short elasto-plastic stage. After reaching the peak load, the load-carrying capacity drops rapidly. When the slenderness ratio ranges from 20 to 60, the initial stiffness gradually decreases, and the post-peak response shows a relatively gradual descending branch, suggesting that the members still retain a certain deformation capacity after instability. When the slenderness ratio exceeds 60, the elastic stiffness is significantly reduced, and the load drops sharply immediately after reaching the peak value, with poor ductility and almost no post-peak load-bearing capacity. This phenomenon suggests that when the slenderness ratio of HGBS columns exceeds 60, second-order lateral effects govern the entire loading process, thereby hindering the full utilization of the material’s strength.

From the above analysis, it can be concluded that the slenderness ratio is a key governing parameter controlling the eccentric compressive performance of HGBS columns. In practical engineering applications, to avoid excessive reduction in load-carrying capacity and potential instability risk, the slenderness ratio of eccentrically loaded HGBS columns should be appropriately limited (recommended λ ≤ 60). The results of the parametric analysis presented in this section provide a useful numerical reference for the engineering design of HGBS columns and the establishment of appropriate slenderness ratio limits.

## 5. Conclusions

This study systematically investigated the mechanical behavior of hollow glued bamboo scrimber (HGBS) columns under eccentric compression through a combination of experimental tests and finite element simulations, with a particular focus on the effects of slenderness ratio on load-carrying capacity, stiffness, and failure modes. Based on the experimental results, model validation, and parametric analysis, the following main conclusions can be drawn:

(1) The comparison between axial compression and eccentric compression tests indicates that the failure mode and ultimate load-carrying capacity of HGBS columns are significantly affected by the load eccentricity. The axially loaded specimens mainly exhibited local crushing failure, indicating that the material strength was relatively well utilized. In contrast, the eccentrically loaded specimens with an eccentricity of 90 mm failed predominantly in a global bending instability mode, accompanied by crushing and wrinkling of bamboo fibers on the compression side. The average ultimate load of the eccentrically loaded specimens was 78.77 kN, which is only about 17% of that of the axially loaded specimens.
(2) A refined finite element model of HGBS columns was developed using ABAQUS. Good agreement was achieved between the numerical simulations and experimental results in terms of failure modes, load–displacement response evolution, and ultimate load-carrying capacity. The relative error in the ultimate load was controlled within 10%, validating the accuracy and reliability of the proposed model in simulating the full-range mechanical behavior of HGBS columns under eccentric compression.
(3) A parametric study on slenderness ratio was conducted based on the validated finite element model, with column heights ranging from 300 mm to 3000 mm, corresponding to slenderness ratios of 8.9–89.1. The results indicate that the slenderness ratio has a decisive influence on the ultimate load-carrying capacity, initial stiffness, and failure behavior of HGBS columns. As the slenderness ratio increases, the ultimate load-carrying capacity exhibits a nonlinear decreasing trend. When the column height increases from 300 mm to 3000 mm, the ultimate load-carrying capacity decreases from 104.17 kN to 28.20 kN, representing a reduction of 72.9%.
(4) All specimens fail through global instability, accompanied by a significant increase in lateral displacement and a reduction in the utilization of the material strength. Therefore, in practical engineering design, the slenderness ratio of eccentrically loaded HGBS columns should be strictly controlled to avoid instability failure caused by insufficient structural stability at high slenderness ratios.

A total of six HGBS column specimens were designed and tested in this study. However, only two loading conditions were considered, namely axial compression and eccentric compression, with each loading condition consisting of three identical specimens. Although these tests provided valuable experimental data for understanding the effects of load eccentricity and slenderness ratio on the mechanical behavior of HGBS columns, the number of specimens was relatively limited and may not fully capture the variability in material and structural performance. The influence of slenderness ratio on the mechanical behavior of HGBS columns was investigated through a parametric numerical study based on a finite element model validated against the experimental results. Therefore, the findings of the present experimental and numerical investigation should be considered preliminary. Future studies are needed to expand the experimental database, incorporate a wider range of influencing parameters, and further explore the theoretical behavior of HGBS columns under eccentric compression, with the aim of establishing reliable design methods for engineering applications.

## Figures and Tables

**Table 1:** Mechanical performance indicators of bamboo scrimber materials.

E_1_ (MPa)	E_2_ (MPa)	E_3_ (MPa)	μ_12_	μ_13_	μ_23_	G_12_ (MPa)	G_13_ (MPa)	G_23_ (MPa)
14,608	1841	2751	0.35	0.38	0.33	823	1347	567

Note: E_1_, E_2_, and E_3_ represent the elastic moduli of the specimens in the longitudinal, tangential, and radial directions, respectively. μ_12_, μ_13_, and μ_23_ represent the Poisson’s ratios in the longitudinal–tangential, longitudinal–radial, and tangential–radial directions, respectively. G_12_, G_13_, and G_23_ represent the shear moduli in the longitudinal–tangential plane, longitudinal–radial plane, and tangential–radial plane, respectively.

**Table 2:** Specific information of the experimental specimens.

Specimen Number	Length (mm)	Slenderness Ratio	Eccentric Angle (°)	e (mm)	Number
1200T20-0-0	1200	35.6	0	0	3
1200T20-0-90			0	90	3

**Table 3:** Experimental results.

Specimen Number	Slenderness Ratio	Peak Load				
		*F*_u1_ (kN)	*F*_u2_ (kN)	*F*_u3_ (kN)	*F*_ue_ (kN)	Cov
1200T20-0-0	35.6	451.98	500.54	421.69	458.07	8.68%
1200T20-0-90		75.97	92.34	68.01	78.77	15.74%

Note: F_u1_, F_u2_, and F_u3_ represent the ultimate bearing capacities of the three HGBS column specimens in the test, and F_ue_ denotes the average ultimate bearing capacity of the three HGBS column specimens.

**Table 4:** Comparison of ultimate load-carrying capacity of HGBS columns between experimental results and finite element analysis.

Specimen Number	*F*_uf_ /(kN)	*F*_u1_ /(kN)	*F*_u2_ /(kN)	*F*_u3_ /(kN)	*F*_ue_ /(kN)	*F*_uf_/*F*_ue_
1200T20-0-0	436.50	451.98	500.54	421.69	458.07	0.95
1200T20-0-90	85.81	75.97	92.34	68.01	78.77	1.09

Note: F_uf_ represents the ultimate load-carrying capacity of the HGBS column specimens obtained from finite element analysis.

**Table 5:** Results of finite element analysis.

Specimen Number	Slenderness Ratio	*F*_uf_ (kN)
300T20-0-90	8.9	104.17
600T20-0-90	17.8	95.08
1200T20-0-90	35.6	85.81
1800T20-0-90	53.5	71.45
2400T20-0-90	64.9	40.58
3000T20-0-90	89.1	28.20

## Data Availability

The original contributions presented in this study are included in the article. Further inquiries can be directed to the corresponding author.
